# The Publication of the State Medical Society Transactions

**Published:** 1873-11

**Authors:** 


### Editorial.

### The Publication of the State Medical Society Transactions.

At a recent meeting of the Medical Society of the County of New York, resolutions were adopted relative to the publication of the Transactions of the State Society. The Society pledged itself to pay its proportion of the expense of the publication providing the Committee on publication should decide to publish them at the expense of the County Societies. We are glad to see that the action of this Society is being endorsed by other Societies, and in this number publish the resolutions adopted at a recent meeting of the Monroe County Society.

Th refusal to publish the transactions of the State Society seems at first a little illiberal, but we think that good will grow out of it, and hope that steps will soon be taken to place the New York State Medical Society upon a basis entirely independent of State control. Now that the Society is alone responsible for the publication of its transactions the members will doubtless take increased pride in making them a model publication. We hope that the Erie County Society will see fit to take some action in the matter.

